# Massive Hematochezia: A Complication of Methamphetamine-Induced Vasculitis Treated by Transcatheter Hemostasis

**DOI:** 10.1155/2011/919236

**Published:** 2011-06-30

**Authors:** Daniel P. Link, Yung-Wei Chi

**Affiliations:** School of Medicine, University of California Davis, Sacramento, CA 95616, USA

## Abstract

A long-term, heavy methamphetamine user with life-threatening rectal hemorrhage was treated with transcatheter occlusion of the bleeding arteries. The bleeding blood vessels were vulnerable submucosal arteries, part of the collateral supply to the distal colon. Visceral arteriography demonstrates severe arterial stenotic lesions of the celiac axis, superior mesenteric artery and the inferior mesenteric artery. Collateral vessels were seen with corkscrew morphology similar to that seen with thromboangiitis obliterans.

## 1. Introduction

Massive Colonic bleeding, Hematochezia, implies an erosion of a mucosal artery, “Dieulafoy lesion." This rare colonic lesion has been described in a few reports dating back to 1985 [1, 2] and is most commonly related to diverticulosis in older patients. The lesion implies an artery that does not taper normal resulting in a vulnerable artery of 1 mm immediately deep to the colonic mucosa [3]. In young adults, vasculitis is a common etiology, older patients atherosclerosis and angiodysplasia are more common [3]. Methamphetamine abuse affects the entire arterial system [4] and may involve the colon and is described as ischemic [5]. There few reports if any demonstrating the effects of such vasculitis on visceral arteriography. There are reports describing transcatheter therapy for hematochezia for lesions called “Dieulafoy" [2, 6]. In this case, whether a true “Dieulafoy lesion" or not, the source of the hemorrhage was submucosal collateral artery, supplying the colon as a result of the severe disease in the visceral arteries.

## 2. Case Presentation

**Figure 1:**
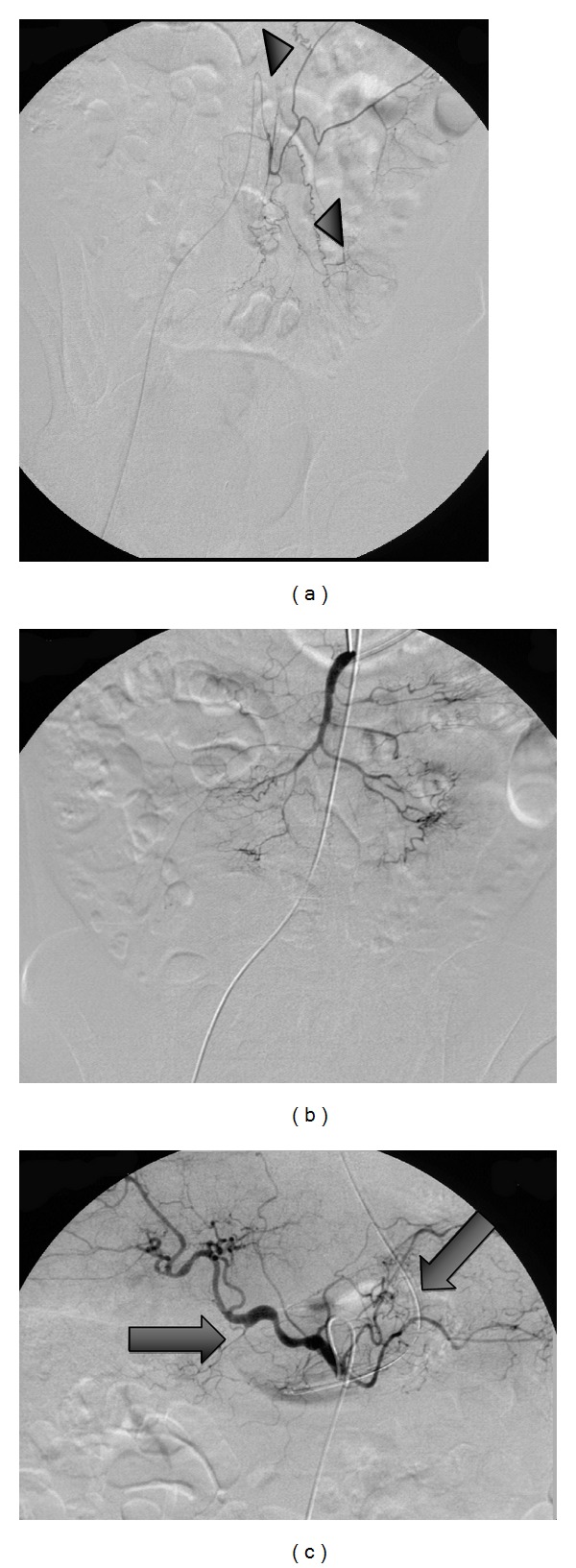
Inferior mesenteric arteriogram: the superior rectal branch is occluded and the sigmoid and left colonic branches are diminished in size. Intramesenteric “corkscrew” collateral arteries (arrow heads), superior mesenteric and celiac axis arteriograms showing diffuse narrowing and focal stenotic lesions, occlusion of the gastroduodenal artery and splenic arteries (arrows).

**Figure 2:**
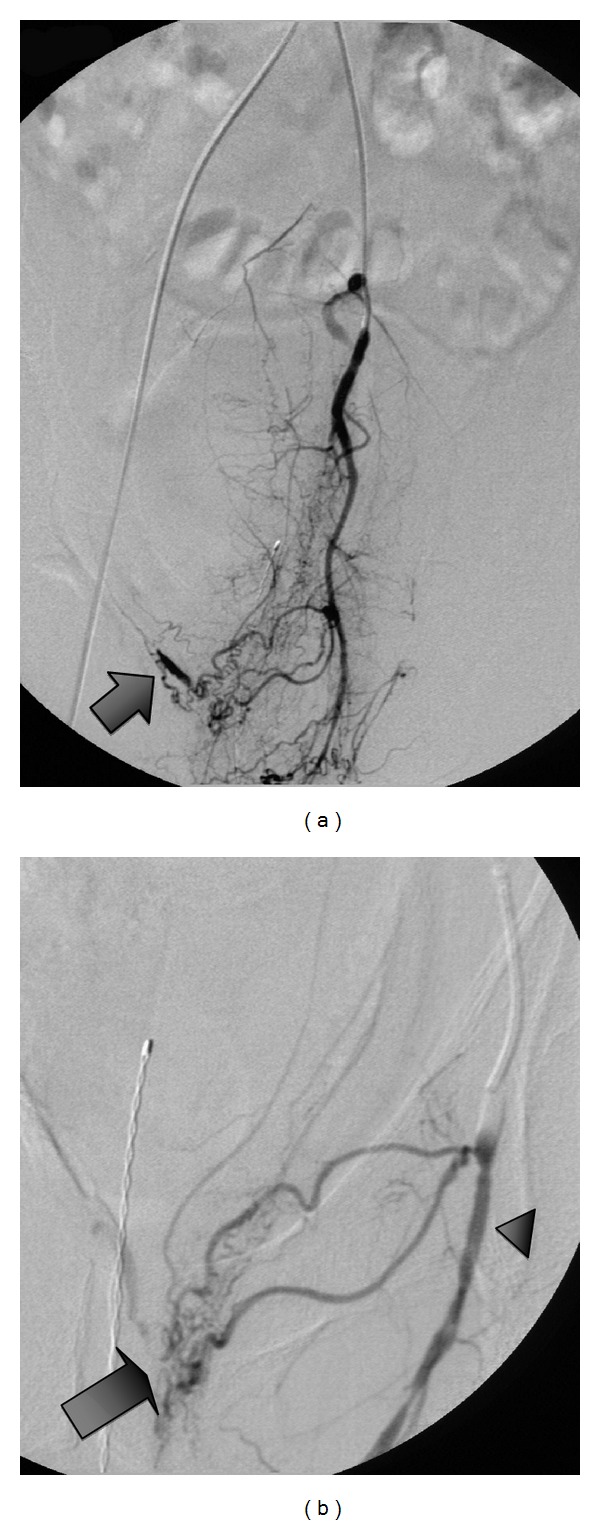
Left internal pudental arteriogram (arrow) (a) bleeding site in the rectum from the inferior rectal branches, *Dieulafoy lesion* (arrow), of the left internal pudental artery (arrow head) (b).

**Figure 3:**
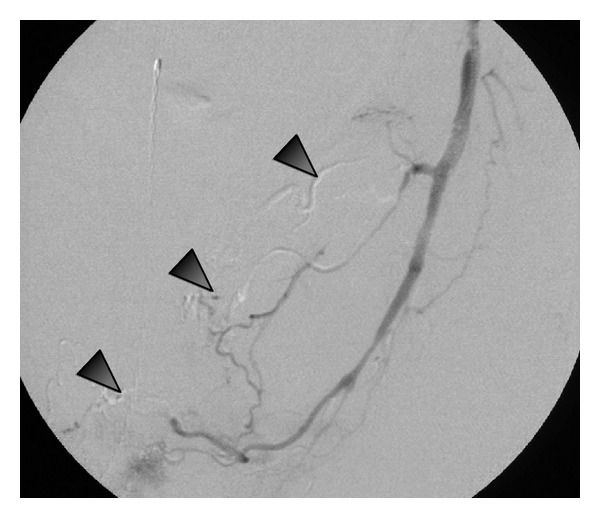
Cast (arrowheads) of n-butyl cyanoacrylate (3 : 1 Eethiodol), occluding the bleeding inferior rectal branches.

45-year-old man with a 20-year history of methamphetamine abuse presented to emergency room with acute sudden onset of abdominal pain and hematochezia. He denied abusing any other substance. He had been seen 3 days earlier for rectal bleeding and local rectal care was advised. He was known to have pulmonary artery hypertension and cardiomyopathy attributed to methamphetamine abuse. A contrast enhanced CT of the abdomen revealed stenotic lesions at the origin of the superior mesenteric and celiac axis of approximately 70%, no bleeding sites or pseudoaneurysms were seen. The visceral stenotic lesions were demonstrated on duplex as well, SMA (pSV at origin 538 cm/sec). Physical exam: tachycardia and orthostatic hypotension. His blood pressure (HR) on admission was 133/66 (74) becoming 82/52 (81) with continued bright red blood, Hct/Hb drop from 49.8% (15 gm/dL) to 35.2% (11.7 gmm/dL despite replacement blood therapy). Endoscopy revealed the stomach and esophagus as normal; with colonoscopy extensive bleeding was encountered obscuring the required detail to identify a specific bleeding source. The bleeding site was identified arteriographically, in the rectum as the branches of the inferior rectal artery (Figure 2), arteriograms of the visceral arteries (Figure 1(a)), the superior mesenteric artery (Figure 1(b)), and celiac axis (Figure 1(c)) revealed diffuse narrowing the visceral vessels with focal areas of occlusion. The inferior mesenteric artery was narrowed with occlusion of the superior rectal artery. “Corkscrew collateral" vessels were seen on the mesentery. In Figure 1(a), the internal pudental artery was catheterized with 4 French angiographic catheter and the inferior rectal arteries selected with a 1.8 F OD microcather (Balt Extrusion, Montmorency, France, *Magic Catheter*), and a 0.008′′ hydrophilic wire guide (*Mirage*, EV3Plymouth, MN, USA). The lesion was treated with 0.5 mL of a 3 : 1 emulsion of n-Butyl Cyanoacrylate (*Trufill*, Cordis Neurovascular, Miami Lakes Florida) and Ethiodol (Nycomed, Inc., USA) (Figure 3). Following the procedure thier immediate relief from the rectal bleeding and the patient continued to improve over the next several days with no further bleeding or signs of intestinal ischemia.

## 3. Discussion

This Presentation includes 3 main teaching points.

1. Vasculitis secondary to methamphetamine is defuse and may be severe.
2. The collateral vascular pattern can resemble thromboangiitis Obliterans.
3. Transcatheter treatment with current technique provides adequate hemostasis.

Bleeding Dieulafoy-type lesions in the rectum have been reported sporadically since 1985 [1]. Since the original description in 1898 by a French Surgeon, the lesion was considered the result of a congenital variant leading to a submucosal artery and has been characterized as a vessel of 1 mm in diameter instead of an arteriole vessel of 0.1 mm. Such large submucosal vessels are vulnerable to bleeding. In this case the large submucosal vessel, Figure 2, was undoubtedly secondary to collateralization to the distal sigmoid and rectum associated with an occlusive vasculitis. (Dieulafoy did not describe a collateral artery, rather a congenital variant.) The findings seen in Figure 1 may be as interesting as the lesion and its treatment, Multiple occlusive lesions and “corkscrew" collateral morphology is a pattern associated with Buerger's Disease [7] which was recently described in the inferior mesenteric artery. In a recent work [8], Corkscrew collateral vessels have seldom been described with other conditions. It is interesting that the “corkscrew" pattern has been duplicated experimentally [9] in a post hind limb occlusion model and thought to be enhanced by placental Growth Factor-1.

The transcatheter treatment and choice of agent was primarily directed by the size of the target vessel. The feeding artery was only able to accept the small diameter, 1.1 Fr, catheter. Such catheter only accepts nonviscous liquids and very small particles, ≪50 microns. The tissue adhesive approach was a method that was able to occlude the network of bleeding rectal vessel, to be a durable occlusion but remain proximal, not inhibiting collateral vessels from the contralateral pelvis.

## 4. Conclusion

Massive bleeding from the rectum is treatable with catheter-based techniques.
